# The Statin-Associated Muscle Symptom Clinical Index (SAMS-CI): Revision for Clinical Use, Content Validation, and Inter-rater Reliability

**DOI:** 10.1007/s10557-017-6723-4

**Published:** 2017-04-18

**Authors:** Robert S Rosenson, Kate Miller, Martha Bayliss, Robert J Sanchez, Marie T Baccara-Dinet, Daniela Chibedi-De-Roche, Beth Taylor, Irfan Khan, Garen Manvelian, Michelle White, Terry A. Jacobson

**Affiliations:** 1 Icahn School of Medicine at Mount Sinai, New York, NY USA; 2 Optum Patient Insights, Lincoln, RI USA; 3 Optum Patient Insights, Johnston, RI USA; 4 Regeneron Pharmaceuticals, Inc., Tarrytown, NY USA; 5 Sanofi, Montpellier, France; 6 Sanofi, Paris, France; 7 Hartford Hospital, Hartford, CT USA; 8 Sanofi, Bridgewater, NJ USA; 9 Emory University, Atlanta, GA USA

**Keywords:** Clinical measurement, Inter-rater reliability, Muscle symptoms, Myalgia, Myopathy, Statin adverse events, Statin intolerance, Statin-associated muscle symptoms

## Abstract

**Purpose:**

The Statin-Associated Muscle Symptom Clinical Index (SAMS-CI) is a method for assessing the likelihood that a patient’s muscle symptoms (e.g., myalgia or myopathy) were caused or worsened by statin use. The objectives of this study were to prepare the SAMS-CI for clinical use, estimate its inter-rater reliability, and collect feedback from physicians on its practical application.

**Methods:**

For content validity, we conducted structured in-depth interviews with its original authors as well as with a panel of independent physicians. Estimation of inter-rater reliability involved an analysis of 30 written clinical cases which were scored by a sample of physicians. A separate group of physicians provided feedback on the clinical use of the SAMS-CI and its potential utility in practice.

**Results:**

Qualitative interviews with providers supported the content validity of the SAMS-CI. Feedback on the clinical use of the SAMS-CI included several perceived benefits (such as brevity, clear wording, and simple scoring process) and some possible concerns (workflow issues and applicability in primary care). The inter-rater reliability of the SAMS-CI was estimated to be 0.77 (confidence interval 0.66–0.85), indicating high concordance between raters. With additional provider feedback, a revised SAMS-CI instrument was created suitable for further testing, both in the clinical setting and in prospective validation studies.

**Conclusions:**

With standardized questions, vetted language, easily interpreted scores, and demonstrated reliability, the SAMS aims to estimate the likelihood that a patient’s muscle symptoms were attributable to statins. The SAMS-CI may support better detection of statin-associated muscle symptoms in clinical practice, optimize treatment for patients experiencing muscle symptoms, and provide a useful tool for further clinical research.

**Electronic supplementary material:**

The online version of this article (doi:10.1007/s10557-017-6723-4) contains supplementary material, which is available to authorized users.

## Introduction

Statins are among the most widely prescribed drugs in the USA [1], and statin-associated muscle symptoms are the most commonly reported adverse events of statin therapy [2]. Approximately 60% of adults who no longer take statins cite muscle pain as the primary reason for discontinuation [3]. Since statins reduce the risk of cardiovascular events, and adherence to statin therapy correlates with reduced cardiovascular mortality, the presence of muscle-related adverse events associated with statin therapy represents a major clinical and public health concern [4, 5]. In fact, one study by Graham et al. indicated that patients with intolerance to statins experienced higher healthcare resource use, higher likelihood of cardiovascular events, and lower likelihood of achieving their low-density lipoprotein cholesterol goal compared to a matched cohort without intolerance to statins [6]. Likewise, a study by Serban and colleagues demonstrated that, among Medicare beneficiaries, intolerance to statins was associated with higher recurrent risk of myocardial infarction and coronary revascularization [7]. The presence of statin-associated muscle symptoms also negatively influences the ability of patients to perform activities of daily living and engage in physical activity, which is concerning because of the strong inverse relationship between physical activity and mortality [8, 9].

**Fig. 1:**
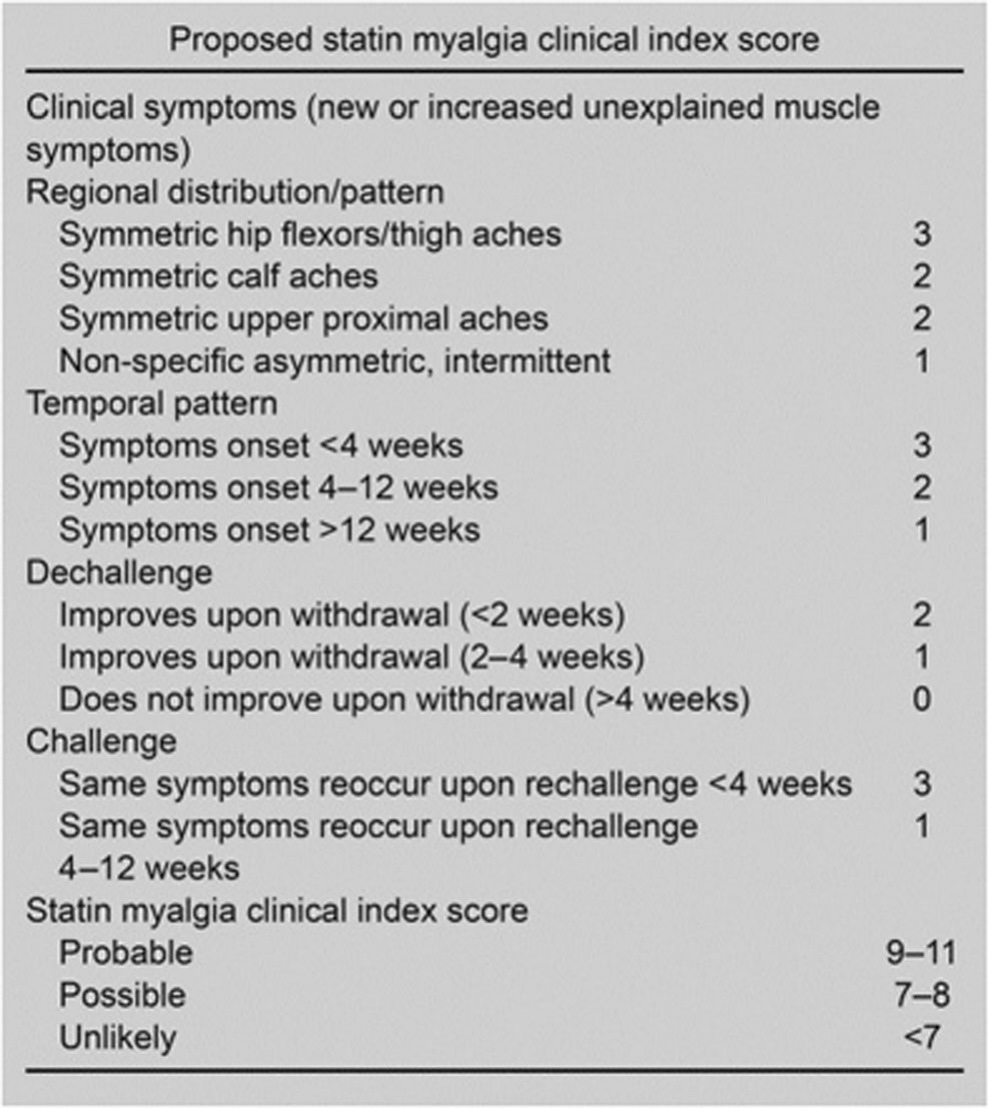
Original Statin Myalgia Clinical Index as proposed by the NLA. Reprinted with permission from Rosenson et al. [2]. *NLA* National Lipid Association, *SMCI* Statin Myalgia Clinical Index, *SAMS-CI* Statin-Associated Muscle Symptom Clinical Index

While no gold standard measure exists for the identification of statin-associated muscle symptoms, progress toward a clear definition came in a 2014 publication from the Statin Muscle Safety Task Force of the National Lipid Association (NLA). In this work, Rosenson et al. [2] defined the spectrum of statin-associated muscle events to include, in increasing order of severity: myalgia (described as flu-like symptoms), myopathy (muscle weakness), myositis (muscle inflammation), myonecrosis (muscle enzyme elevation or increase in creatine kinase), and clinical rhabdomyolysis. To date, there are no standardized measurement instruments to accurately diagnose statin-associated muscle symptoms. Therefore, in order to determine the likelihood that muscle-related effects are attributed to statin use, the NLA Statin Muscle Safety Task Force proposed a new methodology for assessing the likelihood that a statin-treated patient’s myalgia or myopathy were caused or worsened by statin use (Fig. 1).

The method was originally referred to as the Statin Myalgia Clinical Index (SMCI), but was re-named the Statin-Associated Muscle Symptom Clinical Index (SAMS-CI) to reflect its breadth of assessing all forms of statin-associated muscle symptoms outlined by the NLA, not just myalgia. The SAMS-CI includes four separate ratings: the first regards the location and patterns of the muscle symptoms and the remaining three address the timing of symptoms relative to starting, stopping (dechallenge), and rechallenging with statins. As with any new measurement tool, documentation of its development, measurement properties, and practical application are required. This paper therefore reports on: (1) preparing the SAMS-CI for clinical use, including documentation of content validity; (2) estimating its inter-rater reliability; and (3) collecting initial feedback from physicians on the potential clinical use of the tool. These efforts are taken to prepare the tool not only for clinical use, but also for research, such as in an ongoing validation study with an independent cohort to establish the SAMS-CI’ predictive validity.

## Methods

**Fig. 2:**
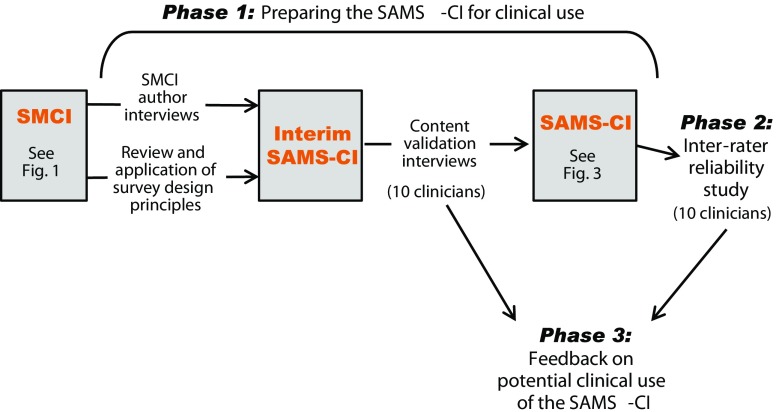
Study phases and SAMS-CI versions

The New England Institutional Review Board approved this study. The three phases of the study are further described in Fig. 2 and consist of: (1) preparing the SAMS-CI for clinical use, (2) estimating its inter-rater reliability, and (3) gathering feedback from clinicians on its potential for clinical use.

### Preparing the SAMS-CI for Clinical Use

**Fig. 3:**
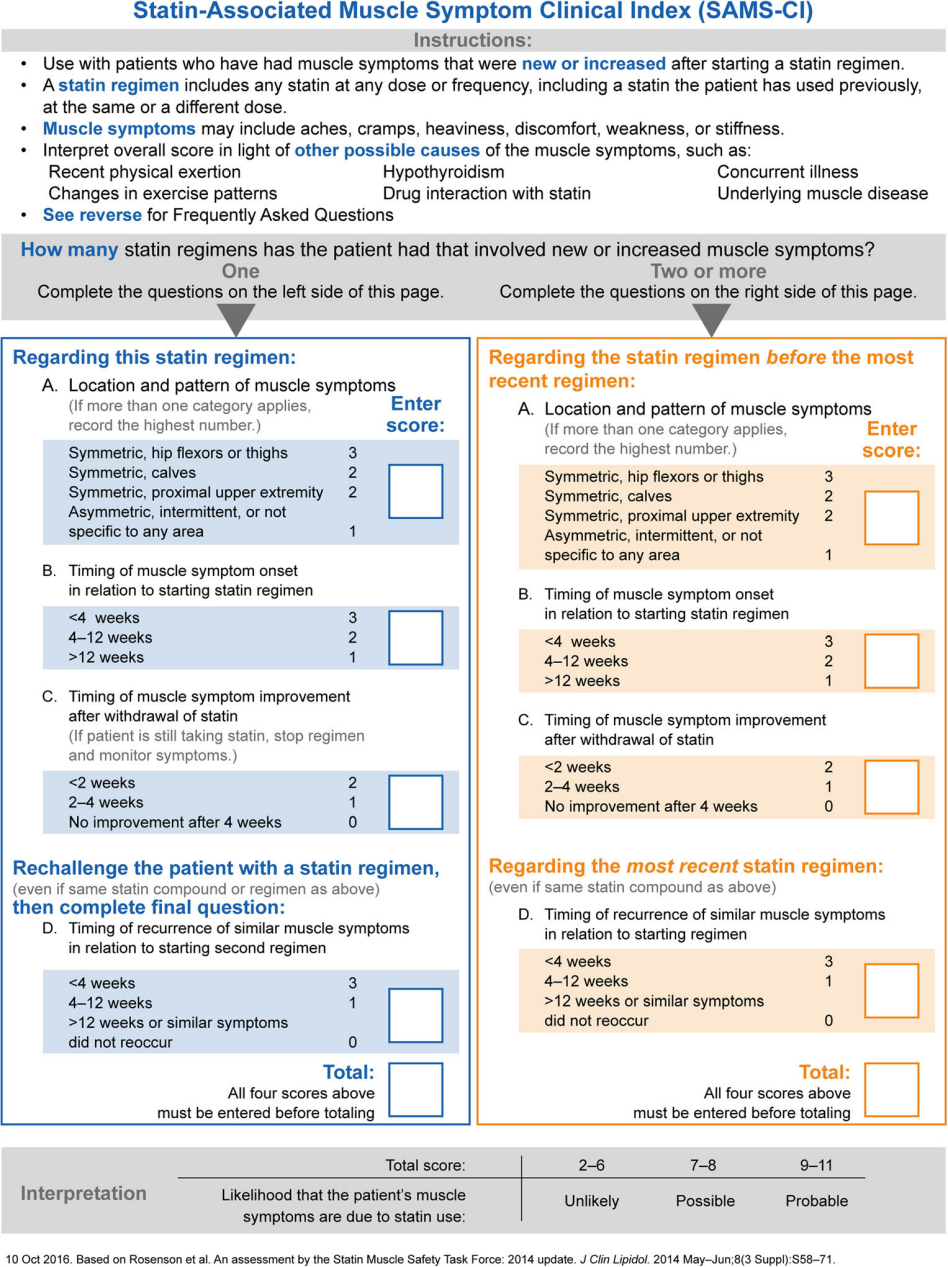
Statin-Associated Muscle Symptoms Clinical Index (SAMS-CI)

For clarity, the original proposed method published by the NLA Statin Muscle Safety Task Force will be referred to as the SMCI (Fig. 1). The intermediate version following author interviews and review will be called the interim SAMS-CI (not shown), and the version prepared for clinical use is denoted as the SAMS-CI (Fig. 3).

Adaptation of the original SMCI for routine clinical use proceeded with three activities, as shown in Fig. 2. First, between October 16 and 22, 2014, hour-long telephone interviews were conducted with a convenience sample of three of the five authors of the SMCI (RSR, BT, and TAJ, also authors of this paper). These semi-structured interviews covered several topics, including the thought process behind each question; the response options, scoring algorithm, and importance weights; the precise definition of certain terms; the type of patient best suited to take the SMCI; and reasons for excluding certain concepts from the SMCI. The interviews were audiotaped and transcribed for analysis.

Second, we conducted a review of the SMCI with regard to everyday use, assessing any missing features and applying survey design principles to the content and layout. Changes to the SMCI following the author interviews and this review resulted in the interim SAMS-CI.

Third, between January 23 and 30, 2015, we conducted content validation interviews of the interim SAMS-CI among 10 clinicians who had no involvement in drafting the SMCI or SAMS-CI. The sample included two primary care physicians and eight cardiologists across the USA (further description available in Online Resource 1). To be included, clinicians needed to write an average of 40 or more statin prescriptions per month, have treated at least 10 patients with statin-associated muscle symptoms in the previous year, and have been in practice post-residency for at least 5 years. These interviews followed a rigorous, standardized qualitative methodology using a semi-structured interview guide to gather detailed information from respondents on the conceptual domains, language clarity, suggested changes, and other reactions to each element of the interim SAMS-CI (instructions, questions, response options, and scoring). The interviews were audiotaped and transcribed for analysis. Respondents were blinded to the identity of the sponsor. We applied the information gathered in these interviews to generate the final SAMS-CI (Fig. 3).

### Estimating Inter-rater Reliability

The next phase of development was to test the reliability of the SAMS-CI. The inter-rater reliability was estimated by the intra-class correlation coefficient (ICC). Using the notation of Shrout and Fleiss [10], we calculated ICC (2,1) as:

$$ \frac{\mathrm{BMS}-\mathrm{EMS}}{\mathrm{BMS}+\left( k-1\right)\mathrm{EMS}+\frac{k\left(\mathrm{JMS}-\mathrm{EMS}\right)}{n}} $$

where BMS is between-case variance, JMS is between-rater variance, EMS is residual (error), *k* is number of raters (e.g., clinicians), and *n* is number of clinical cases. A 95% confidence interval was constructed around the ICC using Fisher’s *z* transformation. In this methodology, statistical power is largely determined by the number of clinical cases rated by each respondent rather than the number of respondents [11]. Thus, we utilized 10 respondents rating 30 case studies, which were sufficiently powered to detect a true ICC above 0.65 with an alpha of 0.05 and a power of 0.85.

Our sample of 10 raters included four primary care physicians and six cardiologists, as shown in Table 1. This sample of clinicians was drawn separately from the content validation sample, but the inclusion criteria were identical. Raters were blinded to the identity of the researchers and sponsor.

**Table 1:** Characteristics of clinicians participating in inter-rater reliability study

Number	Primary practice	Years in practice (post-residency)	Statin prescriptions written per month	Number of statin-associated muscle symptoms cases/year	Region of USA	Gender
1	Primary care	20–30	40–60	20–50	East coast	Female
2	Primary care	10–20	40–60	10–20	East coast	Male
3	Cardiology	10–20	40–60	20–50	South	Male
4	Cardiology	10–20	60+	50+	South	Male
5	Cardiology	10–20	40–60	10–20	South	Male
6	Primary care	10–20	60+	20–50	East coast	Male
7	Primary care	10–20	60+	10–20	East coast	Female
8	Cardiology	10–20	60+	10–20	West coast	Male
9	Cardiology	5–10	40–60	10–20	East coast	Female
10	Cardiology	5–10	60+	10–20	West coast	Male

Each case presented a hypothetical patient with hypercholesterolemia, and the set of cases was designed to cover the full range of possible scores on the SAMS-CI. We used an Excel function to randomly draw 10 clinical states from each of the three possible ratings: “probable,” “possible,” and “unlikely,” as defined by the total SAMS-CI scores. The “probable” vignettes were randomly drawn from among the 14 ways to score “probable” from the SAMS-CI items, for a sampling fraction of 71%. Likewise, the “possible” vignettes were randomly drawn from the 35 “possible” states, for a sampling fraction of 29%. Lastly, the “unlikely” vignettes were randomly drawn from the 59 “unlikely” states, for a sampling fraction of 17%. In all cases, the hypothetical patient had experienced two or more previous episodes of muscle symptoms with statin use. Table 2 contains a sample clinical case from the study, Online Resource 2 presents a screenshot of the SAMS-CI as it appeared to study participants, and Online Resource 3 details all of the clinical cases included in the study.

**Table 2:** Sample clinical case

Labs	
Total cholesterol	205 mg/dL
Triglycerides	125 mg/dL
LDL-C	140 mg/dL
HDL-C	50 mg/dL
Glucose	108 mg/dL
AST	63 u/L (10–30 u/L)
ALT	50 u/L (6–40 u/L)
CPK	50 u/L
Medication	
Losartan	100 mg qd
Amlodipine	10 mg
HCTZ	25 mg
ASA	325 mg
Exam	
Height	5’2”
Weight	160 lbs.

A 70-year-old female presents to the lipid clinic upon referral by her internist for management of dyslipidemia. Her past medical history includes hypertension and a transient ischemic attack. She was started on atorvastatin 40 mg 6 months ago and during the first 2 weeks of therapy, she noticed bilateral upper arm pain and weakness. After stopping the statin, her pain stopped 4 weeks later. Two months ago she was started on rosuvastatin 5 mg every other day but her upper arm pain, which she describes as very similar to her previous symptoms, returned after 1 week of treatment

*ALT* alanine aminotransferase, *ASA* aspirin, *AST* aspartate aminotransferase, *CPK* creatine phosphokinase, *HCTZ* hydrochlorothiazide, *HDL-C* high-density lipoprotein cholesterol, *LDL-C* low-density lipoprotein cholesterol

All cases were developed by a practicing cardiologist (otherwise unaffiliated with this study), who wrote each case to fit the target SAMS-CI score as selected above. The cases were reviewed and edited by two measurement scientists, a clinical nurse, and a copyeditor.

The 30 cases were programmed into an online survey system [Qualtrics.com; Provo, UT, USA], and raters completed the SAMS-CI for all the cases at their convenience within a 2-week period between May 18 and June 1, 2015. The order of presentation of cases was randomized by rater and they were required to provide a rating for every case.

### Collecting Feedback on Potential Clinical Use of the SAMS-CI

In the third phase, the 20 clinicians who participated in the content validation (*n* = 10) and inter-rater reliability (*n* = 10) studies also gave input on the feasibility of using the SAMS-CI in their practice (Fig. 2). They reported on which staff they thought could administer the SAMS-CI to patients, how the SAMS-CI might be integrated with electronic medical systems, and any potential barriers inhibiting its use.

The content validation interview respondents addressed this topic at the end of the in-depth instrument review. The inter-rater reliability respondents addressed the topic during a 30-min telephone interview conducted after they had completed the 30 clinical cases online. These interviews followed a rigorous, standardized qualitative methodology using a semi-structured interview guide, and were audiotaped and transcribed for analysis.

## Results

### Preparing the SAMS-CI for Clinical Use

The revisions to the original SMCI were based on the three sources of information described above: author interviews, review with regard to clinical use, and content validation interviews. Key changes included the addition of a large, visually salient scoring system; revision of question and response option text for simplicity, clarity, and descriptive value; and addition of instructions regarding the overall instrument and certain questions.

Crucially, formatting changes were also made to clarify which questions applied to which muscle symptom episodes. In the SMCI, the patient is assumed to have had a minimum of two previous episodes of muscle symptoms, yet the SAMS-CI should also be relevant for patients who have had only one previous episode, perhaps arising in the primary care setting. In this case, as seen in the finalized SAMS-CI for clinical use shown in Fig. 3, questions A through C could be completed initially, and D could be completed after a statin rechallenge. To accommodate this likely clinical situation, the SAMS-CI is presented in two distinct columns side by side, with slightly different versions for the case of one previous episode of muscle symptoms versus two or more episodes.

### Estimating Inter-Rater Reliability

All 10 raters scored all 30 clinical cases, resulting in 300 SAMS-CI ratings in total with no missing data. Table 3 shows that, among the 30 clinical cases, on average 8.6 of the 10 raters calculated the correct SAMS-CI rating of “probable,” “possible”, or “unlikely”. This average was slightly higher for the extremes—9.0 for “probable” and 8.9 for “unlikely”—and slightly lower for the middle case of “possible” (7.9). The estimated ICC of the SAMS-CI was 0.77, with a 95% confidence interval of 0.66 to 0.85.

**Table 3:** Number of raters correctly classifying clinical cases as “probable,” “possible,” or “unlikely”

Clinical case number	Correct values^a^		Number of raters selecting the correct rating	
	Total score	Rating		
1	11	Probable	10	Average number of raters selecting “probable” correctly: 9.0
2	10		7	
3	10		10	
4	10		10	
5	9		9	
6	9		10	
7	9		10	
8	9		9	
9	9		8	
10	9		7	
11	8	Possible	6	Average number of raters selecting “possible” correctly: 7.9
12	8		8	
13	8		9	
14	8		7	
15	7		6	
16	7		9	
17	7		7	
18	7		9	
19	7		8	
20	7		10	
21	6	Unlikely	9	Average number of raters selecting “unlikely” correctly: 8.9
22	6		8	
23	6		8	
24	6		10	
25	6		8	
26	6		8	
27	5		9	
28	5		10	
29	5		9	
30	4		10	
Average number of raters selecting ratings correctly:			8.6	

^a^The clinical cases were written to produce these “correct” ratings. The word “correct” is used here for clarity, but these are more precisely termed “target” ratings because of the inevitable possibility that the clinical cases were themselves in some way misleading

### Feedback on Potential Clinical Use of the SAMS-CI

All 20 clinicians interviewed regarding the SAMS-CI found it to be clear and brief enough for use in clinical practice, and 12 reported that they would use the SAMS-CI in their own clinical practice. Considerations in favor of using the SAMS-CI in routine clinical practice mentioned by cardiologists and primary care physicians included the following:

- Brief and clear wording and scoring processes.
- Ability of non-physician staff such as nurses or physician assistants to complete it with patients.
- Usefulness in the context of patient counseling, such as triggering a conversation about alternate causes, building the patient’s confidence in the clinical assessment, and educating patients generally about the relationship between statins and muscle symptoms.

Clinicians also cited some possible concerns about routine use:

- Fitting the SAMS-CI into constrained workflows and brief patient visits.
- Concern that the SAMS-CI assesses just one aspect of statin intolerance (the experience of muscle symptoms), and must be interpreted in the context of other clinical information, such as recent physical exertion or hypothyroidism.
- Need for information from two previous episodes of muscle symptoms to calculate a score.

Clinicians also expressed interest in embedding the SAMS-CI into electronic medical record systems, which would streamline the administration of the tool and centralize the results for access by clinical staff.

## Discussion

The SAMS-CI offers a framework for attributing muscle symptoms to statin use. The initial form of the instrument, the SMCI, had not been previously optimized for routine clinical use. This mixed-method study revised the SMCI to produce a new version (the SAMS-CI) ready for use and further testing, both in clinical practice and in research on its predictive validity with real patients. Methods in this study included qualitative research on content validation and a quantitative study of inter-rater reliability. Qualitative results supported the content of the SAMS-CI, and the inter-rater reliability of the SAMS-CI was estimated to be 0.77 (above the customary minimum ICC threshold of 0.7 [12]), indicating strong correspondence between raters. This study also elicited several potential strengths and weaknesses of the SAMS-CI as integrated into clinical care.

Although muscle symptoms often co-occur with statin use, statins are not necessarily their cause. Both the ODYSSEY ALTERNATIVE and GAUSS-3 studies [13, 14] recruited patients with a strong history of muscle-related statin intolerance and used a complex study design in order to enhance the selection of a “truly” statin-intolerant patient population. The ODYSSEY ALTERNATIVE study started with a placebo run-in period, during which patients who experienced muscle-related adverse events were excluded, followed by a randomized controlled trial including a statin control arm. Of the 47 patients who failed to complete the placebo run-in, 48.9% had at least one skeletal muscle event related to placebo and were excluded from the study. The GAUSS-3 study used a two-stage approach using a first-step cross-over procedure to allow identification of patients who developed muscle symptoms during atorvastatin administration, but not during placebo administration and vice-versa. During this cross-over procedure, 26.5% of patients discontinued for intolerable muscle symptoms with placebo (but not atorvastatin) and did not enter the second step of the study.

These two examples demonstrate that reported muscle symptoms are not always related to statin use and illustrate how difficult it is to properly identify the statin intolerance population. These rechallenge studies provide further evidence that the predominant cause of statin intolerance may be the “nocebo” effect, which is totally dependent on patient awareness of a treatment and its potential adverse effects [4, 15]. They may also arise from unrelated factors such as coincident underlying pathologies involving the musculoskeletal system, changes in activity routines, or psychological determinants [15].

The need for tools to identify statin intolerance is rapidly growing. Recently, two manuscripts were published which created an algorithm to identify statin-associated muscle symptoms from claims databases and/or electronic medical records [16, 17]. Furthermore, a patient-level tool, which allows patients to start a dialog about statin-associated muscle symptoms with healthcare providers, has also been developed [18]; and finally, the American College of Cardiology has developed a mobile application to identify statin-associated muscle symptoms. In our study, the SAMS-CI was developed to assist clinicians’ estimates of the likelihood that myalgia or myopathy are attributable to statins. The current study represents the first important step on the road to final validation.

A next step in this process will be to collect evidence about the performance of the SAMS-CI with actual statin patients in practice. Further research can establish the positive and negative predictive values of the SAMS-CI to proactively identify cases of statin-associated muscle symptoms, thereby averting unnecessary rechallenges. While the SAMS-CI has not yet been used in research contexts, it was developed in part from muscle symptom assessment protocols used in two recent clinical trials. The Effect of Statins on Skeletal Muscle Function study [19] used a dechallenge-rechallenge protocol in statin-naive patients, and the Coenzyme Q10 in Statin Myopathy Study [20] used a double-blind, placebo-controlled, randomized, cross-over protocol in patients with a previous history of statin-associated muscle symptoms. In these trials, 30–50% of patients with supposed statin-associated muscle symptoms actually had non-specific muscle pain (pain on placebo). Consequently, as a first step toward validation, we are carrying out a post hoc reanalysis of data from these two trials. Although the SAMS-CI did not exist at the time of these studies, enough detailed data was collected on each patient at baseline to complete the SAMS-CI retrospectively. These scores will then be compared to the patients’ ultimate outcomes in the studies, to estimate the SAMS-CI’ ability to predict statin-associated muscle symptoms among patients on statins as well as to identify patients who experienced muscle symptoms on placebo. These results may suggest further revision of the instrument before a dedicated, prospective study is carried out.

## Conclusions

Attributing muscle symptoms to statin use has long been identified in the literature as a difficult problem to measure, standardize, and quantify. The SAMS-CI resolves some of these issues through the use of standard questions with vetted language, easily interpreted scores, and proven inter-rater reliability. As revised for clinical use, the SAMS-CI may support better detection of statin-associated muscle symptoms in clinical practice, better-optimized treatment for patients experiencing muscle symptoms, and stronger measurement in clinical research. Forthcoming research will seek to validate the SAMS-CI in a larger population and establish its predictive validity.

## Electronic supplementary material

Online Resource 1 (PDF 15.5 kb)

Online Resource 2 (PDF 40.5 kb)

Online Resource 3 (PDF 208 kb)
