# Supplementary material for: The Statin-Associated Muscle Symptom Clinical Index (SAMS-CI): Revision for Clinical Use, Content Validation, and Inter-rater Reliability
Source: Cardiovasc Drugs Ther. 2017 Apr 18;31(2):179–86. doi: 10.1007/s10557-017-6723-4 (PMC5427100; doi:10.1007/s10557-017-6723-4)
Supplement: Supplementary file 1 — (PDF 15.5 kb) [file 10557_2017_6723_MOESM1_ESM.pdf]

Online Resource 1: Characteristics of clinicians participating in content validation interviews

n	Primary practice	Years in practice (post-residency)	Statin prescriptions written per month	# of statin-associated muscle symptoms cases/ year	Region of USA	Gender
1	Cardiology	5–10	60+	50+	East coast	Male
2	Cardiology	10–20	40–60	20–50	Midwest	Female
3	Cardiology	10–20	40–60	10–20	East coast	Male
4	Cardiology	20–30	60+	20–50	East coast	Male
5	Cardiology	20–30	40–60	20–50	West coast	Male
6	Cardiology	30+	40–60	10–20	South	Male
7	Primary care	20–30	60+	10–20	East coast	Female
8	Primary care	10–20	40–60	10–20	West coast	Female
9	Cardiology	10–20	40–60	20–50	West coast	Male
10	Cardiology	5–10	60+	20–50	South	Male
