# Supplementary material for: The Statin-Associated Muscle Symptom Clinical Index (SAMS-CI): Revision for Clinical Use, Content Validation, and Inter-rater Reliability
Source: Cardiovasc Drugs Ther. 2017 Apr 18;31(2):179–86. doi: 10.1007/s10557-017-6723-4 (PMC5427100; doi:10.1007/s10557-017-6723-4)
Supplement: Supplementary file 2 — (PDF 40.5 kb) [file 10557_2017_6723_MOESM2_ESM.pdf]

Online Resource 2. Screenshot of SAMS-CI as presented in inter-rater reliability study

Regarding the statin regimen *before* the most recent regimen:

A. Location and pattern of muscle symptoms
(If more than one category applies, record the highest number.)

☐	Symmetric, hip flexors or thighs	3
☐	Symmetric, calves	2
☐	Symmetric, proximal upper extremity	2
☐	Asymmetric, intermittent, or not specific to any area	1

☐

B. Timing of muscle symptom onset in relation to starting statin regimen

☐	< 4 weeks	3
☐	4 - 12 weeks	2
☐	>12 weeks	1

☐

C. Timing of muscle symptom improvement after withdrawal of statin

☐	< 2 weeks	2
☐	2 - 4 weeks	1
☐	No improvement after 4 weeks	0

☐

Regarding the most recent statin regimen (even if same statin compound as above):

D. Timing of recurrence of similar muscle symptoms in relation to starting second regimen

☐	< 4 weeks	3
☐	4 - 12 weeks	1
☐	>12 weeks or similar symptoms did not reoccur	0

☐
