# Supplementary material for: The Statin-Associated Muscle Symptom Clinical Index (SAMS-CI): Revision for Clinical Use, Content Validation, and Inter-rater Reliability
Source: Cardiovasc Drugs Ther. 2017 Apr 18;31(2):179–86. doi: 10.1007/s10557-017-6723-4 (PMC5427100; doi:10.1007/s10557-017-6723-4)
Supplement: Supplementary file 3 — (PDF 208 kb) [file 10557_2017_6723_MOESM3_ESM.pdf]

Online Resource 3. Clinical vignettes as presented to the participants

Welcome to the study and thank you for participating!

We are testing the Statin-Associated Muscle Symptom Clinical Index (SAMS-CI), a new questionnaire that assesses the muscle symptoms patients may experience when they take statins. The SAMS-CI uses standardized items and scoring to assess the likelihood that the muscle symptoms are attributable to the statin use rather than some other cause.

We will present you with 30 brief clinical vignettes describing a statin patient who has muscle symptoms, and ask you to score each one with the SAMS-CI. Please feel free to complete them at your convenience over the two-week study period, but we ask that you complete no more than ten per day. After you are finished scoring all the vignettes, we will contact you again to schedule a 30-minute phone call to discuss your impressions of the SAMS-CI.

Thanks again, and we hope you enjoy participating.

Instructions

1. Read each vignette carefully. Some of the vignettes will appear very similar, but they all do differ.
2. On the same page as each vignette, score the four SAMS-CI questions by selecting the response for each that fits best in your judgment.
3. When you are finished scoring, press “Next.” The next screen will present the final score and the likelihood that the muscle symptoms were caused by the statin: “Probably,” “Possibly,” or “Unlikely.”
4. Please take note of the SAMS-CI result and whether it agrees with your own clinical judgment of the case. We will ask about this in the telephone interview.
5. Please do not complete more than ten vignettes in one day.
6. In this study, a “statin regimen” includes any statin at any dose or frequency, including a compound the patient has used previously, at the same or a different dose.
7. In this study, “muscle symptoms” may include aches, cramps, heaviness, discomfort, weakness, or stiffness.
8. Please refer to the FAQ page for further information and clarification.

Case Vignette 1

MK underwent a CABG three months ago following a 95% LAD lesion with a V-fib arrest. He is doing well s/p CABG and has resumed exercise without cardiac symptoms. Prior to discharge from the hospital he was started on atorvastatin 80 mg, and approximately 3 weeks after starting the medication he noticed profound heaviness in both his upper legs and was unable to walk up his stairs at home. He was instructed to stop the statin immediately and lab tests revealed no significant elevation in his CPK. After discontinuing the statin, his upper leg heaviness resolved within one week and he was able to resume walking up the stairs at home without difficulty. One month ago he was started on rosuvastatin 5 mg, and he again developed the profound bilateral thigh heaviness, which resolved a week after stopping the medication.

Labs:

Total cholesterol	380 mg/dL
Triglycerides	100 mg/dL
HDL-C	30 mg/dL
LDL-C	330 mg/dL
T4	7.0 ug/dL
TSH	2.0 uIU/mL
CPK	100 mg/dL
ALT	24 u/L

Other Medications:

- Aspirin 81 mg qd
- Clopidogrel Bisulfate 37.5 mg qd
- Ezetimibe 10 mg qd
- Metoprolol Tartrate Oral 25 mg qd
- Niacin 750 mg qd

Exam:

BP 134/76 | Pulse 62 | Resp 16 | Ht 6' 1" | Wt 168 lb | BMI 22.17 kg/m2 | SpO2 99%.

Regarding the statin regimen *before* the most recent regimen:

A. Location and pattern of muscle symptoms
(If more than one category applies, record the highest number.)
- ☐ Symmetric, hip flexors or thighs — 3
- ☐ Symmetric, calves — 2
- ☐ Symmetric, proximal upper extremity — 2
- ☐ Asymmetric, intermittent, or not specific to any area — 1

☐

B. Timing of muscle symptom onset in relation to starting statin regimen
- ☐ < 4 weeks — 3
- ☐ 4 - 12 weeks — 2
- ☐ >12 weeks — 1

☐

C. Timing of muscle symptom improvement after withdrawal of statin
- ☐ < 2 weeks — 2
- ☐ 2 - 4 weeks — 1
- ☐ No improvement after 4 weeks — 0

☐

Regarding the most recent statin regimen (even if same statin compound as above):

D. Timing of recurrence of similar muscle symptoms in relation to starting second regimen
- ☐ < 4 weeks — 3
- ☐ 4 - 12 weeks — 1
- ☐ >12 weeks or similar symptoms did not reoccur — 0

☐

Vignette number	Target scores on individual items A	B	C	D	Target total score	Target rating
1	3	3	2	3	11	Probable

Case Vignette 2

A 68-year-old woman with type 2 diabetes mellitus presents to the lipid clinic with statin intolerance. She has tried atorvastatin and rosuvastatin, but after each therapy she stopped due to profound muscle weakness. She describes her muscle weakness as localized to both her thighs and she is unable to climb stairs or stand up without assistance. At initial treatment with atorvastatin the bilateral upper thigh weakness began within the first 1-2 weeks of initiating therapy and resolved about 3-4 weeks after stopping the statin. After starting treatment with rosuvastatin, her symptoms resumed within 4 weeks. Her CPK levels have always been normal.

Labs:

Total cholesterol	250 mg/dL
Triglycerides	100 mg/dL
HDL-C	40 mg/dL
LDL-C	190 mg/dL
Glucose	128 mg/dL
A1c	6.9%

Other Medications:

- Ezetimibe 10 mg qd
- Metformin 1500 mg/day
- Pioglitazone 30 mg qd
- Synthetic thyroid replacement 0.25 mg qd

Regarding the statin regimen *before* the most recent regimen:

A. Location and pattern of muscle symptoms
(If more than one category applies, record the highest number.)

- ☐ Symmetric, hip flexors or thighs 3
- ☐ Symmetric, calves 2
- ☐ Symmetric, proximal upper extremity 2
- ☐ Asymmetric, intermittent, or not specific to any area 1

☐

B. Timing of muscle symptom onset in relation to starting statin regimen

- ☐ < 4 weeks 3
- ☐ 4 - 12 weeks 2
- ☐ >12 weeks 1

☐

C. Timing of muscle symptom improvement after withdrawal of statin

- ☐ < 2 weeks 2
- ☐ 2 - 4 weeks 1
- ☐ No improvement after 4 weeks 0

☐

Regarding the most recent statin regimen (even if same statin compound as above):

D. Timing of recurrence of similar muscle symptoms in relation to starting second regimen

- ☐ < 4 weeks 3
- ☐ 4 - 12 weeks 1
- ☐ >12 weeks or similar symptoms did not reoccur 0

☐

Vignette number	Target scores on individual items A	B	C	D	Target total score	Target rating
2	3	3	1	3	10	Probable

Case Vignette 3

A 55-year-old Hispanic woman is referred to your lipid clinic for evaluation and treatment of her dyslipidemia. She recently had a stent placed in the right coronary artery and subsequently developed unstable angina. She was started on atorvastatin 80 mg in the hospital and two months after discharge she followed up at the lipid clinic complaining of recently developed weakness in her hips and difficulty getting out of her chair. On physical exam she was noted to have bilateral hip flexor weakness. Her dose of atorvastatin was discontinued and her weakness in her hips resolved within three days. Three weeks ago she was restarted on only atorvastatin 10 mg, and presents today for an urgent visit because she again has bilateral hip flexor weakness and pain with walking.

Labs:

Total cholesterol	260 mg/dL
Triglycerides	300 mg/dL
HDL-C	32 mg/dL
LDL-C	168 mg/dL
CPK	89 u/L

Other Medications:

- Clopidogril 75 mg qd
- Aspirin 81 mg qd
- Ramipril 5 mg qd

Exam:

BP 120/70 | Ht 5'2" | Wt 130 lb | Waist Circumference 36"

Regarding the statin regimen *before* the most recent regimen:

A. Location and pattern of muscle symptoms
(If more than one category applies, record the highest number.)
- ☐ Symmetric, hip flexors or thighs — 3
- ☐ Symmetric, calves — 2
- ☐ Symmetric, proximal upper extremity — 2
- ☐ Asymmetric, intermittent, or not specific to any area — 1

☐

B. Timing of muscle symptom onset in relation to starting statin regimen
- ☐ < 4 weeks — 3
- ☐ 4 - 12 weeks — 2
- ☐ >12 weeks — 1

☐

C. Timing of muscle symptom improvement after withdrawal of statin
- ☐ < 2 weeks — 2
- ☐ 2 - 4 weeks — 1
- ☐ No improvement after 4 weeks — 0

☐

Regarding the most recent statin regimen (even if same statin compound as above):

D. Timing of recurrence of similar muscle symptoms in relation to starting second regimen
- ☐ < 4 weeks — 3
- ☐ 4 - 12 weeks — 1
- ☐ >12 weeks or similar symptoms did not reoccur — 0

☐

Vignette number	Target scores on individual items A	B	C	D	Target total score	Target rating
3	3	2	2	3	10	Probable

Case Vignette 4

A 35-year-old female presents to the lipid clinic upon referral by her OB/Gyn physician for evaluation of severe hypercholesterolemia. Her past medical history is unremarkable. Both her parents died with premature CHD (father age 58 and mother age 57). She had difficulty with fertility and, with therapy, delivered healthy triplets. During pregnancy, she had gestational diabetes. She was started on simvastatin 20 mg six months ago and during the first two weeks of therapy noted bilateral shoulder pain at rest with soreness to touch. Her pain increased with movement. After stopping the statin her shoulder pain stopped within the first few days. Two months ago she was started on rosuvastatin 5 mg every other day, but her bilateral shoulder pain returned after three weeks of treatment.

Labs:

Total cholesterol	345 mg/dL
Triglycerides	125 mg/dL
LDL-C	300 mg/dL
HDL-C	20 mg/dL
Glucose	108 mg/dL
AST	23 u/L (10-30 u/L)
ALT	30 u/L (6-40 u/L)
CPK	50 u/L

Exam:

Ht 5’6” | Wt 198 lbs

Regarding the statin regimen *before* the most recent regimen:

A. Location and pattern of muscle symptoms
(If more than one category applies, record the highest number.)

- ☐ Symmetric, hip flexors or thighs 3
- ☐ Symmetric, calves 2
- ☐ Symmetric, proximal upper extremity 2
- ☐ Asymmetric, intermittent, or not specific to any area 1

☐

B. Timing of muscle symptom onset in relation to starting statin regimen

- ☐ < 4 weeks 3
- ☐ 4 - 12 weeks 2
- ☐ >12 weeks 1

☐

C. Timing of muscle symptom improvement after withdrawal of statin

- ☐ < 2 weeks 2
- ☐ 2 - 4 weeks 1
- ☐ No improvement after 4 weeks 0

☐

Regarding the most recent statin regimen (even if same statin compound as above):

D. Timing of recurrence of similar muscle symptoms in relation to starting second regimen

- ☐ < 4 weeks 3
- ☐ 4 - 12 weeks 1
- ☐ >12 weeks or similar symptoms did not reoccur 0

☐

Vignette number	Target scores on individual items A		B	C	D	Target total score	Target rating
4	2	Upper prox	3	2	3	10	Probable

Case Vignette 5

A 75-year-old female presents to the lipid clinic upon referral by her internist for evaluation of severe hypercholesterolemia. Her past medical history includes rheumatoid arthritis. She is presently on methotrexate and NSAIDS for her RA. She was started on simvastatin 40 mg six months ago and during the first two weeks of therapy she noticed pain and weakness in both her hips, which increased with walking. After stopping the statin, her hip weakness and pain stopped within the first few days. Two months ago she was started on rosuvastatin 5 mg every other day but her bilateral hip weakness, which she describes as very similar to her previous symptoms, returned after eight weeks of treatment.

Labs:

Total cholesterol	285 mg/dL
Triglycerides	125 mg/dL
LDL-C	200 mg/dL
HDL-C	60 mg/dL
Glucose	108 mg/dL
AST	23 u/L (10-30 u/L)
ALT	30 u/L (6-40 u/L)
CPK	50 u/L

Exam:

Ht 5’2” | Wt 111 lbs

Regarding the statin regimen *before* the most recent regimen:

A. Location and pattern of muscle symptoms
(If more than one category applies, record the highest number.)

☐ Symmetric, hip flexors or thighs	3	☐
☐ Symmetric, calves	2	
☐ Symmetric, proximal upper extremity	2	
☐ Asymmetric, intermittent, or not specific to any area	1	

B. Timing of muscle symptom onset in relation to starting statin regimen

☐ < 4 weeks	3	☐
☐ 4 - 12 weeks	2	
☐ >12 weeks	1	

C. Timing of muscle symptom improvement after withdrawal of statin

☐ < 2 weeks	2	☐
☐ 2 - 4 weeks	1	
☐ No improvement after 4 weeks	0	

Regarding the most recent statin regimen (even if same statin compound as above):

D. Timing of recurrence of similar muscle symptoms in relation to starting second regimen

☐ < 4 weeks	3	☐
☐ 4 - 12 weeks	1	
☐ >12 weeks or similar symptoms did not reoccur	0	

Vignette number	Target scores on individual items A	B	C	D	Target total score	Target rating
5	3	3	2	1	9	Probable

Case Vignette 6

A 55-year-old African-American man was referred to your lipid clinic for evaluation and treatment of dyslipidemia. He recently had a stent placed in the left coronary artery after an anterior wall MI. His most recent blood pressure was 180/100 mm Hg and his medications were adjusted to add amlodipine 10 mg. He was started on atorvastatin 80 mg in the hospital and two weeks after discharge he followed up at the lipid clinic complaining of difficulty climbing stairs and severe discomfort and weakness in both hips that was worse than his usual hip discomfort due to osteoarthritis. On physical exam he was noted to have bilateral hip flexor weakness. His atorvastatin was discontinued with the discomfort persisting for another 6-8 weeks. Three weeks ago he was restarted on only atorvastatin 10 mg, and presents today for an urgent visit because he again has bilateral hip flexor weakness and pain with walking.

Labs:

Total cholesterol	217 mg/dL
Triglycerides	100 mg/dL
HDL-C	32 mg/dL
LDL-C	165 mg/dL
CPK	150 u/L

Other Medications:

- Amlodipine 10 mg qd
- Clopidogril 75 mg qd
- Aspirin 81 mg qd
- Ramipril 5 mg qd
- Metaprolol XL 50 mg qd

Exam:

Ht 6'2" | Wt 230 lbs | Waist circumference 36".

Regarding the statin regimen *before* the most recent regimen:

A. Location and pattern of muscle symptoms
(If more than one category applies, record the highest number.)
- ☐ Symmetric, hip flexors or thighs 3
- ☐ Symmetric, calves 2
- ☐ Symmetric, proximal upper extremity 2
- ☐ Asymmetric, intermittent, or not specific to any area 1

☐

B. Timing of muscle symptom onset in relation to starting statin regimen
- ☐ < 4 weeks 3
- ☐ 4 - 12 weeks 2
- ☐ >12 weeks 1

☐

C. Timing of muscle symptom improvement after withdrawal of statin
- ☐ < 2 weeks 2
- ☐ 2 - 4 weeks 1
- ☐ No improvement after 4 weeks 0

☐

Regarding the most recent statin regimen (even if same statin compound as above):

D. Timing of recurrence of similar muscle symptoms in relation to starting second regimen
- ☐ < 4 weeks 3
- ☐ 4 - 12 weeks 1
- ☐ >12 weeks or similar symptoms did not reoccur 0

☐

Vignette number	Target scores on individual items A	B	C	D	Target total score	Target rating
6	3	3	0	3	9	Probable

Case Vignette 7

A 58-year-old woman with type 2 diabetes mellitus presents to the lipid clinic for the management of dyslipidemia. She has tried atorvastatin 10 mg qd but she developed muscle weakness localized to both her thighs and she was unable to climb stairs or stand up without assistance. The bilateral upper thigh weakness began six weeks after initiating therapy and resolved two weeks after stopping the statin. She was restarted on the atorvastatin 10 mg on Monday, Wednesday and Friday along with ezetimibe 10 mg, but her bilateral hip and thigh weakness resumed within a few days. Her CPK levels have always been normal.

Labs:

Total cholesterol	250 mg/dL
Triglycerides	100 mg/dL
HDL-C	40 mg/dL
LDL-C	190 mg/dL
Glucose	128 mg/dL
A1c	6.9%

Other Medications:

Ezetimibe 10 mg qd
Metformin 1500 mg/day
Pioglitazone 30 mg qd
Synthetic thyroid replacement 0.25 mg qd

Regarding the statin regimen *before* the most recent regimen:

A. Location and pattern of muscle symptoms
(If more than one category applies, record the highest number.)

- ☐ Symmetric, hip flexors or thighs — 3
- ☐ Symmetric, calves — 2
- ☐ Symmetric, proximal upper extremity — 2
- ☐ Asymmetric, intermittent, or not specific to any area — 1

☐

B. Timing of muscle symptom onset in relation to starting statin regimen

- ☐ < 4 weeks — 3
- ☐ 4 - 12 weeks — 2
- ☐ >12 weeks — 1

☐

C. Timing of muscle symptom improvement after withdrawal of statin

- ☐ < 2 weeks — 2
- ☐ 2 - 4 weeks — 1
- ☐ No improvement after 4 weeks — 0

☐

Regarding the most recent statin regimen (even if same statin compound as above):

D. Timing of recurrence of similar muscle symptoms in relation to starting second regimen

- ☐ < 4 weeks — 3
- ☐ 4 - 12 weeks — 1
- ☐ >12 weeks or similar symptoms did not reoccur — 0

☐

Vignette number	Target scores on individual items A	B	C	D	Target total score	Target rating
7	3	2	1	3	9	Probable

Case Vignette 8

A 65-year-old African-American man is referred to your lipid clinic for evaluation and treatment of dyslipidemia and hypertension. He recently had a stent placed in the left coronary artery after an anterior wall MI. He was started on atorvastatin 80 mg in the hospital and two weeks after discharge he followed up at the lipid clinic complaining of severe cramps in both calves, especially at night. His atorvastatin was discontinued and he continued to complain of leg cramps for another 2-3 weeks. Three weeks ago he was restarted on only atorvastatin 10 mg, and presents today for an urgent visit because he again has severe leg cramps.

Labs:

Total cholesterol	253 mg/dL
Triglycerides	100 mg/dL
HDL-C	38 mg/dL
LDL-C	195 mg/dL
CPK	300 u/L

Other Medications:

- Clopidogril 75 mg qd
- Aspirin 81 mg qd
- Ramipril 5 mg qd
- Metaprolol XL 50 mg qd

Exam: Ht 6’2” | Wt 230 lbs | Waist circumference 36” | BP 180/100 mm Hg

Regarding the statin regimen *before* the most recent regimen:

A. Location and pattern of muscle symptoms
(If more than one category applies, record the highest number.)

- ☐ Symmetric, hip flexors or thighs 3
- ☐ Symmetric, calves 2
- ☐ Symmetric, proximal upper extremity 2
- ☐ Asymmetric, intermittent, or not specific to any area 1

B. Timing of muscle symptom onset in relation to starting statin regimen

- ☐ < 4 weeks 3
- ☐ 4 - 12 weeks 2
- ☐ >12 weeks 1

C. Timing of muscle symptom improvement after withdrawal of statin

- ☐ < 2 weeks 2
- ☐ 2 - 4 weeks 1
- ☐ No improvement after 4 weeks 0

Regarding the most recent statin regimen (even if same statin compound as above):

D. Timing of recurrence of similar muscle symptoms in relation to starting second regimen

- ☐ < 4 weeks 3
- ☐ 4 - 12 weeks 1
- ☐ >12 weeks or similar symptoms did not reoccur 0

Vignette number	Target scores on individual items					Target total score	Target rating
	A		B	C	D		
8	2	Calves	3	1	3	9	Probable

Case Vignette 9

A 70-year-old female presents to the lipid clinic upon referral by her internist for management of dyslipidemia. Her past medical history includes hypertension and a TIA. She was started on atorvastatin 40 mg six months ago and during the first two weeks of therapy she noticed bilateral upper arm pain and weakness. After stopping the statin her pain stopped four weeks later. Two months ago she was started on rosuvastatin 5 mg every other day but her upper arm pain, which she describes as very similar to her previous symptoms, returned after one week of treatment.

Labs:

Total cholesterol	205 mg/dL
Triglycerides	125 mg/dL
LDL-C	140 mg/dL
HDL-C	50 mg/dL
Glucose	108 mg/dL
AST	63 u/L (10-30 u/L)
ALT	50 u/L (6-40 u/L)
CPK	50 u/L

Medications

- Losartan 100 mg qd
- Amlodipine 10 mg
- HCTZ 25 mg
- ASA 325 mg

Exam:

Ht 5'2" | Wt 160 lbs.

Regarding the statin regimen *before* the most recent regimen:

A. Location and pattern of muscle symptoms
(If more than one category applies, record the highest number.)
- ☐ Symmetric, hip flexors or thighs 3
- ☐ Symmetric, calves 2
- ☐ Symmetric, proximal upper extremity 2
- ☐ Asymmetric, intermittent, or not specific to any area 1

☐

B. Timing of muscle symptom onset in relation to starting statin regimen
- ☐ < 4 weeks 3
- ☐ 4 - 12 weeks 2
- ☐ >12 weeks 1

☐

C. Timing of muscle symptom improvement after withdrawal of statin
- ☐ < 2 weeks 2
- ☐ 2 - 4 weeks 1
- ☐ No improvement after 4 weeks 0

☐

Regarding the most recent statin regimen (even if same statin compound as above):

D. Timing of recurrence of similar muscle symptoms in relation to starting second regimen
- ☐ < 4 weeks 3
- ☐ 4 - 12 weeks 1
- ☐ >12 weeks or similar symptoms did not reoccur 0

☐

Vignette number	Target scores on individual items					Target total score	Target rating
	A		B	C	D		
9	2	Upper prox	3	1	3	9	Probable

Case Vignette 10

A 55-year-old Hispanic woman is referred to your lipid clinic for evaluation and treatment of her dyslipidemia. She recently had a stent placed in the right coronary artery after she developed unstable angina. She was started on atorvastatin 80 mg in the hospital and two months after discharge she followed up at the lipid clinic complaining of recently developed heaviness and discomfort in both arms up to her shoulders. Her dose of atorvastatin was discontinued and her weakness in her arms resolved within three days. Three weeks ago she was restarted on atorvastatin 10 mg, and presents today for an urgent visit because she again has bilateral pain and weakness in both upper arms.

Labs:

Total cholesterol	260 mg/dL
Triglycerides	300 mg/dL
HDL-C	32 mg/dL
LDL-C	168 mg/dL
CPK	89 u/L

Other Medications:

Clopidogril 75 mg qd
Aspirin 81 mg qd
Ramipril 5 mg qd

Exam:

Ht 5’2” | Wt 130 lbs | Waist circumference 37” | BP 120/70

Regarding the statin regimen *before* the most recent regimen:

A. Location and pattern of muscle symptoms
(If more than one category applies, record the highest number.)
- ☐ Symmetric, hip flexors or thighs 3
- ☐ Symmetric, calves 2
- ☐ Symmetric, proximal upper extremity 2
- ☐ Asymmetric, intermittent, or not specific to any area 1

☐

B. Timing of muscle symptom onset in relation to starting statin regimen
- ☐ < 4 weeks 3
- ☐ 4 - 12 weeks 2
- ☐ >12 weeks 1

☐

C. Timing of muscle symptom improvement after withdrawal of statin
- ☐ < 2 weeks 2
- ☐ 2 - 4 weeks 1
- ☐ No improvement after 4 weeks 0

☐

Regarding the most recent statin regimen (even if same statin compound as above):

D. Timing of recurrence of similar muscle symptoms in relation to starting second regimen
- ☐ < 4 weeks 3
- ☐ 4 - 12 weeks 1
- ☐ >12 weeks or similar symptoms did not reoccur 0

☐

Vignette number	Target scores on individual items					Target total score	Target rating
	A		B	C	D		
10	2	Upper prox	2	2	3	9	Probable

Case Vignette 11

TP underwent a CABG three months ago following a 95% LAD, 90% proximal RCA, and 85% left circumflex lesions. He is doing well s/p CABG and has resumed exercise without cardiac symptoms. Prior to discharge from the hospital he was started on atorvastatin 80 mg, and approximately three weeks after starting the medication he noticed cramps and weakness in both calves and was unable to walk up his stairs at home. He was instructed to stop the statin immediately and lab tests revealed no significant elevation in his CPK. After discontinuing the statin he continued to have leg cramps for the next three months. Less than one month ago he was started on rosuvastatin 5 mg, and he again developed the profound leg cramps. He now returns to the clinic off statin therapy and is unwilling to consider another statin or red yeast rice.

Labs:

Total cholesterol	380 mg/dL
Triglycerides	100 mg/dL
HDL-C	30 mg/dL
LDL-C	330 mg/dL
T4	7.0 ug/dL
TSH	2.0 uIU/mL
CPK	100 mg/dL
ALT	24 U/L

Other Medications:

- Aspirin 81 mg qd
- Clopidogrel Bisulfate (Plavix Oral), 37.5 mg qd
- Ezetimibe (Zetia) 10 mg qd
- Metoprolol Tartrate Oral, 25 mg bid
- Niacin 750 mg qd

Exam:

BP 134/76 | Pulse 62 | Resp 16 | Ht 185.4 cm (6' 1") | Wt 76.204 kg (168 lb) | BMI 22.17 kg/m2 | SpO2 99%

Regarding the statin regimen *before* the most recent regimen:

A. Location and pattern of muscle symptoms
(If more than one category applies, record the highest number.)
- ☐ Symmetric, hip flexors or thighs 3
- ☐ Symmetric, calves 2
- ☐ Symmetric, proximal upper extremity 2
- ☐ Asymmetric, intermittent, or not specific to any area 1

☐

B. Timing of muscle symptom onset in relation to starting statin regimen
- ☐ < 4 weeks 3
- ☐ 4 - 12 weeks 2
- ☐ >12 weeks 1

☐

C. Timing of muscle symptom improvement after withdrawal of statin
- ☐ < 2 weeks 2
- ☐ 2 - 4 weeks 1
- ☐ No improvement after 4 weeks 0

☐

Regarding the most recent statin regimen (even if same statin compound as above):

D. Timing of recurrence of similar muscle symptoms in relation to starting second regimen
- ☐ < 4 weeks 3
- ☐ 4 - 12 weeks 1
- ☐ >12 weeks or similar symptoms did not reoccur 0

☐

Vignette number	Target scores on individual items A		B	C	D	Target total score	Target rating
11	2	Calves	3	0	3	8	Possible

Case Vignette 12

A 63-year-old woman with type 2 diabetes mellitus presents to the lipid clinic with statin intolerance. She has tried atorvastatin and rosuvastatin, but after each therapy she stopped due to profound bilateral shoulder weakness. She describes her muscle weakness as localized to both shoulders and she is unable to perform usual housework such as cleaning or making the bed. With atorvastatin, the bilateral shoulder weakness began within the first one to two weeks of initiating therapy and did not resolve until four to five months after stopping the statin. After restarting statin treatment with rosuvastatin, her symptoms resumed within four weeks. Her CPK levels have always been normal.

Labs:

Total cholesterol	210 mg/dL
Triglycerides	100 mg/dL
HDL-C	40 mg/dL
LDL-C	150 mg/dL
Glucose	128 mg/dL
A1c	6.9%

Other Medications:

- Ezetimibe 10 mg qd
- Metformin 1500 mg/day
- Pioglitazone 30 mg qd
- Synthetic thyroid replacement 0.25 mg qd

Regarding the statin regimen *before* the most recent regimen:

A. Location and pattern of muscle symptoms
(If more than one category applies, record the highest number.)

☐ Symmetric, hip flexors or thighs	3	☐
☐ Symmetric, calves	2	
☐ Symmetric, proximal upper extremity	2	
☐ Asymmetric, intermittent, or not specific to any area	1	

B. Timing of muscle symptom onset in relation to starting statin regimen

☐ < 4 weeks	3	☐
☐ 4 - 12 weeks	2	
☐ >12 weeks	1	

C. Timing of muscle symptom improvement after withdrawal of statin

☐ < 2 weeks	2	☐
☐ 2 - 4 weeks	1	
☐ No improvement after 4 weeks	0	

Regarding the most recent statin regimen (even if same statin compound as above):

D. Timing of recurrence of similar muscle symptoms in relation to starting second regimen

☐ < 4 weeks	3	☐
☐ 4 - 12 weeks	1	
☐ >12 weeks or similar symptoms did not reoccur	0	

Vignette number	Target scores on individual items A		B	C	D	Target total score	Target rating
12	2	Upper prox	3	0	3	8	Possible

Case Vignette 13

A 65-year-old Hispanic woman recently had a stent placed in the right coronary artery after she developed unstable angina. She was started on atorvastatin 80 mg in the hospital and six weeks after discharge she followed up at the lipid clinic complaining of recently developed weakness in her left hip. The left hip weakness occurred after walking and grocery shopping but resolved after rest. Her dose of atorvastatin was discontinued and the weakness in her left hip continued for a week. Three weeks ago she was restarted on atorvastatin 10 mg, and presents today for an urgent visit because her left hip flexor weakness has reoccurred, especially with walking.

Labs:

Total cholesterol	192 mg/dL
Triglycerides	300 mg/dL
HDL-C	32 mg/dL
LDL-C	100 mg/dL
CPK	89 u/L

Other Medications:

- Clopidogril 75 mg qd
- Aspirin 81 mg qd
- Ramipril 5 mg qd

Regarding the statin regimen *before* the most recent regimen:

A. Location and pattern of muscle symptoms
(If more than one category applies, record the highest number.)

- ☐ Symmetric, hip flexors or thighs — 3
- ☐ Symmetric, calves — 2
- ☐ Symmetric, proximal upper extremity — 2
- ☐ Asymmetric, intermittent, or not specific to any area — 1

B. Timing of muscle symptom onset in relation to starting statin regimen

- ☐ < 4 weeks — 3
- ☐ 4 - 12 weeks — 2
- ☐ >12 weeks — 1

C. Timing of muscle symptom improvement after withdrawal of statin

- ☐ < 2 weeks — 2
- ☐ 2 - 4 weeks — 1
- ☐ No improvement after 4 weeks — 0

Regarding the most recent statin regimen (even if same statin compound as above):

D. Timing of recurrence of similar muscle symptoms in relation to starting second regimen

- ☐ < 4 weeks — 3
- ☐ 4 - 12 weeks — 1
- ☐ >12 weeks or similar symptoms did not reoccur — 0

Vignette number	Target scores on individual items				Target total score	Target rating
	A	B	C	D		
13	1	2	2	3	8	Possible

Case Vignette 14

A 45-year-old female presents to the lipid clinic upon referral by her internist for evaluation of severe hypercholesterolemia. Her past medical history is unremarkable. She was started on simvastatin 20 mg six months ago and after two months of therapy her mild bilateral hip discomfort increased with walking. Prior to starting the medication, she reported pain at 3 out of 10 on the pain scale, but after starting simvastatin, her pain increased to 7 out of 10. After stopping the statin, her hip pain was reduced ten days later to 4 out of 10. Two months ago she was started on rosuvastatin 5 mg every other day but her bilateral hip pain recently increased to 8 out of 10.

Labs:

Total cholesterol	345 mg/dL
Triglycerides	125 mg/dL
LDL-C	300 mg/dL
HDL-C	20 mg/dL
Glucose	108 mg/dL
AST	23 u/L (10-30 u/L)
ALT	30 u/L (6-40 u/L)
CPK	50 u/L

Exam:
Ht 5’6” | Wt 198 lbs

Regarding the statin regimen *before* the most recent regimen:

A. Location and pattern of muscle symptoms
(If more than one category applies, record the highest number.)
- ☐ Symmetric, hip flexors or thighs 3
- ☐ Symmetric, calves 2
- ☐ Symmetric, proximal upper extremity 2
- ☐ Asymmetric, intermittent, or not specific to any area 1

☐

B. Timing of muscle symptom onset in relation to starting statin regimen
- ☐ < 4 weeks 3
- ☐ 4 - 12 weeks 2
- ☐ >12 weeks 1

☐

C. Timing of muscle symptom improvement after withdrawal of statin
- ☐ < 2 weeks 2
- ☐ 2 - 4 weeks 1
- ☐ No improvement after 4 weeks 0

☐

Regarding the most recent statin regimen (even if same statin compound as above):

D. Timing of recurrence of similar muscle symptoms in relation to starting second regimen
- ☐ < 4 weeks 3
- ☐ 4 - 12 weeks 1
- ☐ >12 weeks or similar symptoms did not reoccur 0

☐

Vignette number	Target scores on individual items				Target total score	Target rating
	A	B	C	D		
14	3	2	2	1	8	Possible

Case Vignette 15

A 75-year-old female presents to the lipid clinic upon referral by her internist for evaluation of severe hypercholesterolemia. She was started on pravastatin 40 mg six months ago and after four months of therapy she noticed pain and weakness in both shoulders and upper arms. After stopping the statin her shoulder pain and weakness persisted for four weeks. Two weeks ago she was restarted on pravastatin 40 mg qd but her bilateral shoulder weakness, which she describes as very similar to her previous symptoms, returned.

Labs:

Total cholesterol	285 mg/dL
Triglycerides	125 mg/dL
LDL-C	200 mg/dL
HDL-C	60 mg/dL
Glucose	108 mg/dL
AST	23 u/L (10-30 u/L)
ALT	30 u/L (6-40 u/L)
CPK	50 u/L

Exam:
Ht 5’2” | Wt 111 lbs.

Regarding the statin regimen *before* the most recent regimen:

A. Location and pattern of muscle symptoms
(If more than one category applies, record the highest number.)
- ☐ Symmetric, hip flexors or thighs 3
- ☐ Symmetric, calves 2
- ☐ Symmetric, proximal upper extremity 2
- ☐ Asymmetric, intermittent, or not specific to any area 1

☐

B. Timing of muscle symptom onset in relation to starting statin regimen
- ☐ < 4 weeks 3
- ☐ 4 - 12 weeks 2
- ☐ >12 weeks 1

☐

C. Timing of muscle symptom improvement after withdrawal of statin
- ☐ < 2 weeks 2
- ☐ 2 - 4 weeks 1
- ☐ No improvement after 4 weeks 0

☐

Regarding the most recent statin regimen (even if same statin compound as above):

D. Timing of recurrence of similar muscle symptoms in relation to starting second regimen
- ☐ < 4 weeks 3
- ☐ 4 - 12 weeks 1
- ☐ >12 weeks or similar symptoms did not reoccur 0

☐

Vignette number	Target scores on individual items					Target total score	Target rating
	A		B	C	D		
15	2	Upper prox	1	1	3	7	Possible

Case Vignette 16

A 55-year-old African-American man recently had a stent placed in the left coronary artery after an anterior wall MI. He was started on atorvastatin 80 mg in the hospital and six weeks after discharge he followed up at the lipid clinic complaining of severe pain and weakness in both shoulders. The symptoms had developed in the week before his visit. His atorvastatin was discontinued with the pain and weakness persisting for another 6-8 weeks. Three weeks ago he was restarted on atorvastatin 10 mg, and presents today for an urgent visit because he again has bilateral shoulder weakness and pain.

Labs:

Total cholesterol	200 mg/dL
Triglycerides	100 mg/dL
HDL-C	52 mg/dL
LDL-C	128 mg/dL
CPK	100 u/L

Other Medications:

- Clopidogril 75 mg qd
- Aspirin 81 mg qd
- Ramipril 5 mg qd
- Metaprolol XL 50 mg qd

Exam:

Ht 6'2" | Wt 230 lbs | Waist circumference 36" | BP 140/85

Regarding the statin regimen *before* the most recent regimen:

A. Location and pattern of muscle symptoms
(If more than one category applies, record the highest number.)

☐	Symmetric, hip flexors or thighs	3	☐
☐	Symmetric, calves	2	
☐	Symmetric, proximal upper extremity	2	
☐	Asymmetric, intermittent, or not specific to any area	1	

B. Timing of muscle symptom onset in relation to starting statin regimen

☐	< 4 weeks	3	☐
☐	4 - 12 weeks	2	
☐	>12 weeks	1	

C. Timing of muscle symptom improvement after withdrawal of statin

☐	< 2 weeks	2	☐
☐	2 - 4 weeks	1	
☐	No improvement after 4 weeks	0	

Regarding the most recent statin regimen (even if same statin compound as above):

D. Timing of recurrence of similar muscle symptoms in relation to starting second regimen

☐	< 4 weeks	3	☐
☐	4 - 12 weeks	1	
☐	>12 weeks or similar symptoms did not reoccur	0	

Vignette number	Target scores on individual items A		B	C	D	Target total score	Target rating
16	2	Upper prox	2	0	3	7	Possible

Case Vignette 17

A 58-year-old woman with type 2 diabetes mellitus presents to the lipid clinic for the management of dyslipidemia. She tried atorvastatin 10 mg qd but she developed muscle weakness localized to both her thighs and she was unable to climb stairs or stand up without assistance. The bilateral upper thigh weakness began three weeks after initiating therapy and continued for six weeks after stopping the statin. She was restarted on the atorvastatin 10 mg on Monday, Wednesday and Friday along with ezetimibe 10 mg, but her bilateral hip and thigh weakness resumed six weeks later. Her CPK levels have always been normal

Labs:

Total cholesterol	250 mg/dL
Triglycerides	100 mg/dL
HDL-C	40 mg/dL
LDL-C	190 mg/dL
Glucose	128 mg/dL
A1c	6.9%

Other Medications:

- Ezetimibe 10 mg qd
- Metformin 1500 mg/day
- Pioglitazone 30 mg qd
- Synthetic thyroid replacement 0.25 mg qd

Regarding the statin regimen *before* the most recent regimen:

A. Location and pattern of muscle symptoms
(If more than one category applies, record the highest number.)

- ☐ Symmetric, hip flexors or thighs 3
- ☐ Symmetric, calves 2
- ☐ Symmetric, proximal upper extremity 2
- ☐ Asymmetric, intermittent, or not specific to any area 1

☐

B. Timing of muscle symptom onset in relation to starting statin regimen

- ☐ < 4 weeks 3
- ☐ 4 - 12 weeks 2
- ☐ >12 weeks 1

☐

C. Timing of muscle symptom improvement after withdrawal of statin

- ☐ < 2 weeks 2
- ☐ 2 - 4 weeks 1
- ☐ No improvement after 4 weeks 0

☐

Regarding the most recent statin regimen (even if same statin compound as above):

D. Timing of recurrence of similar muscle symptoms in relation to starting second regimen

- ☐ < 4 weeks 3
- ☐ 4 - 12 weeks 1
- ☐ >12 weeks or similar symptoms did not reoccur 0

☐

Vignette number	Target scores on individual items A	B	C	D	Target total score	Target rating
17	3	3	0	1	7	Possible

Case Vignette 18

A 65-year-old man is referred to your lipid clinic for evaluation and treatment of dyslipidemia. He recently had a stent placed in the left coronary artery after an anterior wall MI. He was started on atorvastatin 80 mg in the hospital and six weeks after discharge he followed up at the lipid clinic complaining of recently developed severe cramps in both upper arms, especially at night. His atorvastatin was discontinued and his arm cramps improved with two days. Six weeks ago he was restarted on atorvastatin 10 mg, and presents today for an urgent visit because he again has severe upper arm cramps.

Labs:

Total cholesterol	178 mg/dL
Triglycerides	100 mg/dL
HDL-C	52 mg/dL
LDL-C	108 mg/dL
CPK	300 u/L

Other Medications:

- Lopidogril 75 mg qd
- Aspirin 81 mg qd
- Ramipril 5 mg qd
- Metaprolol XL 50 mg qd

Regarding the statin regimen *before* the most recent regimen:

A. Location and pattern of muscle symptoms
(If more than one category applies, record the highest number.)

- ☐ Symmetric, hip flexors or thighs — 3
- ☐ Symmetric, calves — 2
- ☐ Symmetric, proximal upper extremity — 2
- ☐ Asymmetric, intermittent, or not specific to any area — 1

☐

B. Timing of muscle symptom onset in relation to starting statin regimen

- ☐ < 4 weeks — 3
- ☐ 4 - 12 weeks — 2
- ☐ >12 weeks — 1

☐

C. Timing of muscle symptom improvement after withdrawal of statin

- ☐ < 2 weeks — 2
- ☐ 2 - 4 weeks — 1
- ☐ No improvement after 4 weeks — 0

☐

Regarding the most recent statin regimen (even if same statin compound as above):

D. Timing of recurrence of similar muscle symptoms in relation to starting second regimen

- ☐ < 4 weeks — 3
- ☐ 4 - 12 weeks — 1
- ☐ >12 weeks or similar symptoms did not reoccur — 0

☐

Vignette number	Target scores on individual items					Target total score	Target rating
	A		B	C	D		
18	2	Upper prox	2	2	1	7	Possible

Case Vignette 19

A 70-year-old female has a past medical history that includes hypertension and a TIA. She was started on atorvastatin 40 mg six months ago and after the first two weeks of therapy she noticed left upper arm pain and weakness intermittently. After stopping the statin her pain stopped one week later. Two months ago she was started on rosuvastatin 5 mg every other day but her upper arm pain, which she describes as very similar to her previous symptoms, returned after six weeks of treatment. She now returns to the clinic for further evaluation.

Labs:

Total cholesterol	205 mg/dL
Triglycerides	125 mg/dL
LDL-C	140 mg/dL
HDL-C	50 mg/dL
Glucose	108 mg/dL
AST	63 u/L (10-30 u/L)
ALT	50 u/L (6-40 u/L)
CPK	50 u/L

Other Medications:

Losartan 100 mg qd
Amlodipine 10 mg qd
HCTZ 25 mg qd
ASA 325 mg qd

Exam:

Ht 5'2" | Wt 160 lbs

Regarding the statin regimen *before* the most recent regimen:

A. Location and pattern of muscle symptoms
(If more than one category applies, record the highest number.)
- ☐ Symmetric, hip flexors or thighs 3
- ☐ Symmetric, calves 2
- ☐ Symmetric, proximal upper extremity 2
- ☐ Asymmetric, intermittent, or not specific to any area 1

☐

B. Timing of muscle symptom onset in relation to starting statin regimen
- ☐ < 4 weeks 3
- ☐ 4 - 12 weeks 2
- ☐ >12 weeks 1

☐

C. Timing of muscle symptom improvement after withdrawal of statin
- ☐ < 2 weeks 2
- ☐ 2 - 4 weeks 1
- ☐ No improvement after 4 weeks 0

☐

Regarding the most recent statin regimen (even if same statin compound as above):

D. Timing of recurrence of similar muscle symptoms in relation to starting second regimen
- ☐ < 4 weeks 3
- ☐ 4 - 12 weeks 1
- ☐ >12 weeks or similar symptoms did not reoccur 0

☐

Vignette number	Target scores on individual items A	B	C	D	Target total score	Target rating
19	1	3	2	1	7	Possible

Case Vignette 20

A 55-year-old Hispanic woman is referred to your lipid clinic for evaluation and treatment of her dyslipidemia. She recently had a stent placed in the right coronary artery after she developed unstable angina. She was started on atorvastatin 80 mg in the hospital and two weeks after discharge she followed up at the lipid clinic complaining of stiffness and cramps in both arms up to her shoulders. Her dose of atorvastatin was discontinued and her weakness in her arms resolved within three days. Three months ago she was restarted on only atorvastatin 10 mg, and denies having musculoskeletal symptoms.

Labs:

Total cholesterol	220 mg/dL
Triglycerides	300 mg/dL
HDL-C	32 mg/dL
LDL-C	128 mg/dL

Other Medications:

Clopidogril 75 mg qd
Aspirin 81 mg qd
Ramipril 5 mg qd

Exam:
Ht 5’2” | Wt 130 lbs | Waist circumference 36” | BP 120/70

Regarding the statin regimen *before* the most recent regimen:

A. Location and pattern of muscle symptoms
(If more than one category applies, record the highest number.)
- ☐ Symmetric, hip flexors or thighs 3
- ☐ Symmetric, calves 2
- ☐ Symmetric, proximal upper extremity 2
- ☐ Asymmetric, intermittent, or not specific to any area 1

☐

B. Timing of muscle symptom onset in relation to starting statin regimen
- ☐ < 4 weeks 3
- ☐ 4 - 12 weeks 2
- ☐ >12 weeks 1

☐

C. Timing of muscle symptom improvement after withdrawal of statin
- ☐ < 2 weeks 2
- ☐ 2 - 4 weeks 1
- ☐ No improvement after 4 weeks 0

☐

Regarding the most recent statin regimen (even if same statin compound as above):

D. Timing of recurrence of similar muscle symptoms in relation to starting second regimen
- ☐ < 4 weeks 3
- ☐ 4 - 12 weeks 1
- ☐ >12 weeks or similar symptoms did not reoccur 0

☐

Vignette number	Target scores on individual items					Target total score	Target rating
	A		B	C	D		
20	2	Upper prox	3	2	0	7	Possible

Case Vignette 21

A 65-year-old African-American woman is referred to your lipid clinic for evaluation and treatment of her dyslipidemia. She recently had a stent placed in the left coronary artery after she developed unstable angina. She was started on atorvastatin 80 mg in the hospital and two weeks after discharge she followed up at the lipid clinic complaining of stiffness and cramps in both thighs and hips. Her dose of atorvastatin was discontinued and her symptoms resolved three months later. Four months ago she started ezetimibe 10 mg qd and has not had a recurrence of symptoms.

Labs:

Total cholesterol	222 mg/dL
Triglycerides	300 mg/dL
HDL-C	42 mg/dL
LDL-C	120 mg/dL
CPK	189 u/L

Other Medications:

Clopidogril 75 mg qd
Aspirin 81 mg qd
Ramipril 5 mg qd

Exam:
Ht 5’2” | Wt 180 lbs |Waist circumference 42” | BP 150/90 mm Hg

Regarding the statin regimen *before* the most recent regimen:

A. Location and pattern of muscle symptoms
(If more than one category applies, record the highest number.)
- ☐ Symmetric, hip flexors or thighs — 3
- ☐ Symmetric, calves — 2
- ☐ Symmetric, proximal upper extremity — 2
- ☐ Asymmetric, intermittent, or not specific to any area — 1

☐

B. Timing of muscle symptom onset in relation to starting statin regimen
- ☐ < 4 weeks — 3
- ☐ 4 - 12 weeks — 2
- ☐ >12 weeks — 1

☐

C. Timing of muscle symptom improvement after withdrawal of statin
- ☐ < 2 weeks — 2
- ☐ 2 - 4 weeks — 1
- ☐ No improvement after 4 weeks — 0

☐

Regarding the most recent statin regimen (even if same statin compound as above):

D. Timing of recurrence of similar muscle symptoms in relation to starting second regimen
- ☐ < 4 weeks — 3
- ☐ 4 - 12 weeks — 1
- ☐ >12 weeks or similar symptoms did not reoccur — 0

☐

Vignette number	Target scores on individual items A	B	C	D	Target total score	Target rating
21	3	3	0	0	6	Unlikely

Case Vignette 22

MP underwent a CABG three months ago following a 95% LAD, 90% proximal RCA, and 85% left circumflex lesions. He is doing well s/p CABG and has resumed exercise without cardiac symptoms. Prior to discharge from the hospital he was started on atorvastatin 80 mg, and approximately six weeks after starting the medication he noticed cramps and weakness in both hips especially after exercise and was unable to walk up his stairs at home. He was instructed to stop the statin immediately and lab tests revealed no significant elevation in his CPK. After discontinuing the statin he continued to have hip cramps for the next month. Three months ago he was started on rosuvastatin 5 mg, and denies hip pain or weakness.

Labs:

Total cholesterol	180 mg/dL
Triglycerides	100 mg/dL
HDL-C	30 mg/dL
LDL-C	130 mg/dL
T4	7.0 ug/dL
TSH	2.0 uIU/mL
CPK	100 mg/dL
ALT	24 U/L

Other Medications:

- Aspirin 81 mg qd
- Clopidogrel Bisulfate (Plavix) 37.5 mg qd
- Ezetimibe 10 mg qd
- Metoprolol Tartrate 25 mg bid
- Niacin 750 mg qd

Exam:

BP 134/76 | Pulse 62 | Resp 16 | Ht 185.4 cm (5'9" | Wt 76.204 kg (168 lb) | BMI 26.17 kg/m2 | SpO2 99%

Regarding the statin regimen *before* the most recent regimen:

A. Location and pattern of muscle symptoms
(If more than one category applies, record the highest number.)
- ☐ Symmetric, hip flexors or thighs 3
- ☐ Symmetric, calves 2
- ☐ Symmetric, proximal upper extremity 2
- ☐ Asymmetric, intermittent, or not specific to any area 1

☐

B. Timing of muscle symptom onset in relation to starting statin regimen
- ☐ < 4 weeks 3
- ☐ 4 - 12 weeks 2
- ☐ >12 weeks 1

☐

C. Timing of muscle symptom improvement after withdrawal of statin
- ☐ < 2 weeks 2
- ☐ 2 - 4 weeks 1
- ☐ No improvement after 4 weeks 0

☐

Regarding the most recent statin regimen (even if same statin compound as above):

D. Timing of recurrence of similar muscle symptoms in relation to starting second regimen
- ☐ < 4 weeks 3
- ☐ 4 - 12 weeks 1
- ☐ >12 weeks or similar symptoms did not reoccur 0

☐

Vignette number	Target scores on individual items A	B	C	D	Target total score	Target rating
22	3	2	1	0	6	Unlikely

Case Vignette 23

A 63-year-old woman with type 2 diabetes mellitus presents to the lipid clinic with question of statin intolerance. She has tried atorvastatin 40 mg, but stopped due to profound bilateral lower leg heaviness and discomfort. The bilateral leg discomfort began two months after initiating therapy and resolved one week after stopping the statin. Her CPK levels have always been normal. She presents in the office today with bilateral leg discomfort beginning four months after initiating therapy with atorvastatin 10 mg.

Labs:

Total cholesterol	210 mg/dL
Triglycerides	100 mg/dL
HDL-C	40 mg/dL
LDL-C	150 mg/dL
Glucose	128 mg/dL
A1c	6.9%

Other Medications:

- Ezetimibe 10 mg qd
- Metformin 1500 mg/day
- Pioglitazone 30 mg qd
- Synthetic thyroid replacement 0.25 mg qd

Regarding the statin regimen *before* the most recent regimen:

A. Location and pattern of muscle symptoms
(If more than one category applies, record the highest number.)
- ☐ Symmetric, hip flexors or thighs — 3
- ☐ Symmetric, calves — 2
- ☐ Symmetric, proximal upper extremity — 2
- ☐ Asymmetric, intermittent, or not specific to any area — 1

B. Timing of muscle symptom onset in relation to starting statin regimen
- ☐ < 4 weeks — 3
- ☐ 4 - 12 weeks — 2
- ☐ >12 weeks — 1

C. Timing of muscle symptom improvement after withdrawal of statin
- ☐ < 2 weeks — 2
- ☐ 2 - 4 weeks — 1
- ☐ No improvement after 4 weeks — 0

Regarding the most recent statin regimen (even if same statin compound as above):

D. Timing of recurrence of similar muscle symptoms in relation to starting second regimen
- ☐ < 4 weeks — 3
- ☐ 4 - 12 weeks — 1
- ☐ >12 weeks or similar symptoms did not reoccur — 0

Vignette number	Target scores on individual items					Target total score	Target rating
	A		B	C	D		
23	2	Calves	2	2	0	6	Unlikely

Case Vignette 24

A 65-year-old Hispanic woman recently had a stent placed in the right coronary artery after she developed unstable angina. She was started on atorvastatin 80 mg in the hospital and four months after discharge she followed up at the lipid clinic complaining of recently developed cramps in both leg calves with difficulty walking. Her dose of atorvastatin was discontinued but leg cramps continued intermittently for an additional four months. Three weeks ago she was restarted on atorvastatin 10 mg, and presents today for an urgent visit because her leg cramps have reoccurred especially with walking.

Labs:

Total cholesterol	192 mg/dL
Triglycerides	300 mg/dL
HDL-C	32 mg/dL
LDL-C	100 mg/dL
CPK	89 u/L

Other Medications:

- Clopidogril 75 mg qd
- Aspirin 81 mg qd
- Ramipril 5 mg qd

Exam:
Ht 5’2” | Wt 130 lbs | Waist circumference 36” | BP 120/70 mm Hg.

Regarding the statin regimen *before* the most recent regimen:

A. Location and pattern of muscle symptoms
(If more than one category applies, record the highest number.)

- ☐ Symmetric, hip flexors or thighs — 3
- ☐ Symmetric, calves — 2
- ☐ Symmetric, proximal upper extremity — 2
- ☐ Asymmetric, intermittent, or not specific to any area — 1

☐

B. Timing of muscle symptom onset in relation to starting statin regimen

- ☐ < 4 weeks — 3
- ☐ 4 - 12 weeks — 2
- ☐ >12 weeks — 1

☐

C. Timing of muscle symptom improvement after withdrawal of statin

- ☐ < 2 weeks — 2
- ☐ 2 - 4 weeks — 1
- ☐ No improvement after 4 weeks — 0

☐

Regarding the most recent statin regimen (even if same statin compound as above):

D. Timing of recurrence of similar muscle symptoms in relation to starting second regimen

- ☐ < 4 weeks — 3
- ☐ 4 - 12 weeks — 1
- ☐ >12 weeks or similar symptoms did not reoccur — 0

☐

Vignette number	Target scores on individual items					Target total score	Target rating
	A		B	C	D		
24	2	Calves	1	0	3	6	Unlikely

Case Vignette 25

A 63-year-old female presents to the lipid clinic with possible statin intolerance. Both her parents died with premature CHD (father age 58 and mother age 57). She has hypertension and smokes two packs of cigarettes per day. She was started on rosuvastatin 20 mg six months ago and after three weeks of therapy she noted isolated left hip and arm pain. Her pain increased with walking or lifting groceries and seemed to improve at rest. After stopping the statin her hip and arm pain stopped ten days later. Four months ago she was started on rosuvastatin 5 mg every other day but her left hip and arm pain returned recently.

Labs:

Total cholesterol	285 mg/dL
Triglycerides	125 mg/dL
LDL-C	200 mg/dL
HDL-C	60 mg/dL
Glucose	108 mg/dL
AST	23 u/L (10-30 u/L)
ALT	30 u/L (6-40 u/L)
CPK	50 u/L

Regarding the statin regimen *before* the most recent regimen:

A. Location and pattern of muscle symptoms
(If more than one category applies, record the highest number.)
- ☐ Symmetric, hip flexors or thighs 3
- ☐ Symmetric, calves 2
- ☐ Symmetric, proximal upper extremity 2
- ☐ Asymmetric, intermittent, or not specific to any area 1

☐

B. Timing of muscle symptom onset in relation to starting statin regimen
- ☐ < 4 weeks 3
- ☐ 4 - 12 weeks 2
- ☐ >12 weeks 1

☐

C. Timing of muscle symptom improvement after withdrawal of statin
- ☐ < 2 weeks 2
- ☐ 2 - 4 weeks 1
- ☐ No improvement after 4 weeks 0

☐

Regarding the most recent statin regimen (even if same statin compound as above):

D. Timing of recurrence of similar muscle symptoms in relation to starting second regimen
- ☐ < 4 weeks 3
- ☐ 4 - 12 weeks 1
- ☐ >12 weeks or similar symptoms did not reoccur 0

☐

Vignette number	Target scores on individual items A	B	C	D	Target total score	Target rating
25	1	3	2	0	6	Unlikely

Case Vignette 26

A 70 -year-old female presents to the lipid clinic upon referral by her internist for evaluation of severe hypercholesterolemia. She was started on pravastatin 40 mg six months ago and after one week of therapy she noticed pain in her left back and shoulder. After stopping the statin her shoulder and back pain persisted for four weeks. Two months ago she was restarted on pravastatin 40 mg qd but her back and shoulder pain has recently returned, which she describes as very similar to her previous symptoms.

Labs:

Total cholesterol	285 mg/dL
Triglycerides	125 mg/dL
LDL-C	200 mg/dL
HDL-C	60 mg/dL
Glucose	108 mg/dL
AST	23 u/L (10-30 u/L)
ALT	30 u/L (6-40 u/L)
CPK	50 u/L

Exam:

Ht 5'2" | Wt 111 lbs

Regarding the statin regimen *before* the most recent regimen:

A. Location and pattern of muscle symptoms
(If more than one category applies, record the highest number.)
- ☐ Symmetric, hip flexors or thighs 3
- ☐ Symmetric, calves 2
- ☐ Symmetric, proximal upper extremity 2
- ☐ Asymmetric, intermittent, or not specific to any area 1

B. Timing of muscle symptom onset in relation to starting statin regimen
- ☐ < 4 weeks 3
- ☐ 4 - 12 weeks 2
- ☐ >12 weeks 1

C. Timing of muscle symptom improvement after withdrawal of statin
- ☐ < 2 weeks 2
- ☐ 2 - 4 weeks 1
- ☐ No improvement after 4 weeks 0

Regarding the most recent statin regimen (even if same statin compound as above):

D. Timing of recurrence of similar muscle symptoms in relation to starting second regimen
- ☐ < 4 weeks 3
- ☐ 4 - 12 weeks 1
- ☐ >12 weeks or similar symptoms did not reoccur 0

Vignette number	Target scores on individual items				Target total score	Target rating
	A	B	C	D		
26	1	3	1	1	6	Unlikely

Case Vignette 27

A 65-year-old African-American man had a stent placed in the left coronary artery after an anterior wall MI. He was started on atorvastatin 80 mg in the hospital and three weeks after discharge he followed up at the lipid clinic complaining of severe pain and weakness in both shoulders. The symptoms developed a few days before his visit. His atorvastatin was discontinued with the pain and weakness persisting for 6-8 weeks. He was restarted on atorvastatin 10 mg and presents today for a six-month follow-up, and he complains of no pain.

Labs:

Total cholesterol	200 mg/dL
Triglycerides	100 mg/dL
HDL-C	52 mg/dL
LDL-C	128 mg/dL
CPK	160 u/L

Other Medications;

- Clopidogril 75 mg qd
- Aspirin 81 mg qd
- Ramipril 5 mg qd
- Metaprolol XL 50 mg qd

Exam:

Ht 6'2" | Wt 230 lbs | Waist circumference 36" | BP 140/87 mm Hg

Regarding the statin regimen *before* the most recent regimen:

A. Location and pattern of muscle symptoms
(If more than one category applies, record the highest number.)

- ☐ Symmetric, hip flexors or thighs 3
- ☐ Symmetric, calves 2
- ☐ Symmetric, proximal upper extremity 2
- ☐ Asymmetric, intermittent, or not specific to any area 1

☐

B. Timing of muscle symptom onset in relation to starting statin regimen

- ☐ < 4 weeks 3
- ☐ 4 - 12 weeks 2
- ☐ >12 weeks 1

☐

C. Timing of muscle symptom improvement after withdrawal of statin

- ☐ < 2 weeks 2
- ☐ 2 - 4 weeks 1
- ☐ No improvement after 4 weeks 0

☐

Regarding the most recent statin regimen (even if same statin compound as above):

D. Timing of recurrence of similar muscle symptoms in relation to starting second regimen

- ☐ < 4 weeks 3
- ☐ 4 - 12 weeks 1
- ☐ >12 weeks or similar symptoms did not reoccur 0

☐

Vignette number	Target scores on individual items					Target total score	Target rating
	A		B	C	D		
27	2	Upper prox	3	0	0	5	Unlikely

Case Vignette 28

A 58-year-old woman with type 2 diabetes mellitus presents to the lipid clinic for the management of dyslipidemia. She tried atorvastatin 10 mg qd but she developed muscle weakness “all over her body” that started a month after initiation of treatment and improved a few days after stopping treatment. The diffuse body weakness stopped about one week after discontinuing the statin. She was restarted on the atorvastatin 10 on Monday, Wednesday and Friday along with ezetimibe 10 mg, and she no longer complains of body weakness after more than 12 weeks of therapy. Her CPK levels have always been normal.

Labs:

Total cholesterol	150 mg/dL
Triglycerides	100 mg/dL
HDL-C	40 mg/dL
LDL-C	90 mg/dL
Glucose	128 mg/dL
A1c	6.9%

Other Medications:

Ezetimibe 10 mg qd
Metformin 1500 mg/day
Pioglitazone 30 mg qd
Synthetic thyroid replacement 0.25 mg qd

Regarding the statin regimen *before* the most recent regimen:

A. Location and pattern of muscle symptoms
(If more than one category applies, record the highest number.)
- ☐ Symmetric, hip flexors or thighs — 3
- ☐ Symmetric, calves — 2
- ☐ Symmetric, proximal upper extremity — 2
- ☐ Asymmetric, intermittent, or not specific to any area — 1

B. Timing of muscle symptom onset in relation to starting statin regimen
- ☐ < 4 weeks — 3
- ☐ 4 - 12 weeks — 2
- ☐ >12 weeks — 1

C. Timing of muscle symptom improvement after withdrawal of statin
- ☐ < 2 weeks — 2
- ☐ 2 - 4 weeks — 1
- ☐ No improvement after 4 weeks — 0

Regarding the most recent statin regimen (even if same statin compound as above):

D. Timing of recurrence of similar muscle symptoms in relation to starting second regimen
- ☐ < 4 weeks — 3
- ☐ 4 - 12 weeks — 1
- ☐ >12 weeks or similar symptoms did not reoccur — 0

Vignette number	Target scores on individual items A	B	C	D	Target total score	Target rating
28	1	2	2	0	5	Unlikely

Case Vignette 29

A 65-year-old African-American man is referred to your lipid clinic for evaluation and treatment of dyslipidemia and hypertension. He previously had a stent placed in the left coronary artery after an anterior wall MI. He was started on atorvastatin 80 mg in the hospital and six months after discharge he followed up at the lipid clinic complaining of recent stiffness in his back and right knee especially at night. His atorvastatin was discontinued and his back and knee stiffness improved within two days. Six weeks ago he was restarted on only atorvastatin 10 mg, and presents today for an urgent visit because he again notes back and knee pain.

Labs:

Total cholesterol	178 mg/dL
Triglycerides	100 mg/dL
HDL-C	52 mg/dL
LDL-C	108 mg/dL
CPK	175 u/L

Other Medications:

- Clopidogril 75 mg qd
- Aspirin 81 mg qd
- Ramipril 5 mg qd
- Metaprolol XL 50 mg qd
- Amlodipine 10mg qd (added today)

Exam:

Ht 6'2" | Wt 230 lbs | Waist circumference 36" | BP 180/100 mm

Regarding the statin regimen *before* the most recent regimen:

A. Location and pattern of muscle symptoms
(If more than one category applies, record the highest number.)
- ☐ Symmetric, hip flexors or thighs 3
- ☐ Symmetric, calves 2
- ☐ Symmetric, proximal upper extremity 2
- ☐ Asymmetric, intermittent, or not specific to any area 1

☐

B. Timing of muscle symptom onset in relation to starting statin regimen
- ☐ < 4 weeks 3
- ☐ 4 - 12 weeks 2
- ☐ >12 weeks 1

☐

C. Timing of muscle symptom improvement after withdrawal of statin
- ☐ < 2 weeks 2
- ☐ 2 - 4 weeks 1
- ☐ No improvement after 4 weeks 0

☐

Regarding the most recent statin regimen (even if same statin compound as above):

D. Timing of recurrence of similar muscle symptoms in relation to starting second regimen
- ☐ < 4 weeks 3
- ☐ 4 - 12 weeks 1
- ☐ >12 weeks or similar symptoms did not reoccur 0

☐

Vignette number	Target scores on individual items				Target total score	Target rating
	A	B	C	D		
29	1	1	2	1	5	Unlikely

Case Vignette 30

A 70-year-old female was referred by her internist for management of dyslipidemia. Her past medical history includes hypertension and a TIA. She was started on atorvastatin 40 mg and after 2 weeks, she noticed left upper arm pain and weakness intermittently. She stopped her atorvastatin but her left arm pain continued for three months. She was started on rosuvastatin 5 mg three months ago and reports at this time no recurrence of her arm pain and weakness.

Labs:

Total cholesterol	205 mg/dL
Triglycerides	125 mg/dL
LDL-C	140 mg/dL
HDL-C	50 mg/dL
Glucose	108 mg/dL
AST	63 u/L (10-30 u/L)
ALT	50 u/L (6-40 u/L)
CPK	50 u/L

Other Medications:

Losartan 100 mg qd
Amlodipine 10 mg qd
HCTZ 25 mg qd
ASA 325 mg qd

Exam:
Ht 5'2" | Wt 160 lbs

Regarding the statin regimen *before* the most recent regimen:

A. Location and pattern of muscle symptoms
(If more than one category applies, record the highest number.)
- ☐ Symmetric, hip flexors or thighs 3
- ☐ Symmetric, calves 2
- ☐ Symmetric, proximal upper extremity 2
- ☐ Asymmetric, intermittent, or not specific to any area 1

B. Timing of muscle symptom onset in relation to starting statin regimen
- ☐ < 4 weeks 3
- ☐ 4 - 12 weeks 2
- ☐ >12 weeks 1

C. Timing of muscle symptom improvement after withdrawal of statin
- ☐ < 2 weeks 2
- ☐ 2 - 4 weeks 1
- ☐ No improvement after 4 weeks 0

Regarding the most recent statin regimen (even if same statin compound as above):

D. Timing of recurrence of similar muscle symptoms in relation to starting second regimen
- ☐ < 4 weeks 3
- ☐ 4 - 12 weeks 1
- ☐ >12 weeks or similar symptoms did not reoccur 0

Vignette number	Target scores on individual items A	B	C	D	Target total score	Target rating
30	1	3	0	0	4	Unlikely
